# Review of Journal of Cardiovascular Magnetic Resonance 2014

**DOI:** 10.1186/s12968-015-0203-4

**Published:** 2015-11-20

**Authors:** D. J. Pennell, A. J. Baksi, S. K. Prasad, C. E. Raphael, P. J. Kilner, R. H. Mohiaddin, F. Alpendurada, S. V. Babu-Narayan, J. Schneider, D. N. Firmin

**Affiliations:** Cardiovascular Biomedical Research Unit, Royal Brompton & Harefield NHS Foundation Trust & Imperial College, Sydney Street, London, SW 3 6NP UK

## Abstract

There were 102 articles published in the Journal of Cardiovascular Magnetic Resonance (JCMR) in 2014, which is a 6 % decrease on the 109 articles published in 2013. The quality of the submissions continues to increase. The 2013 JCMR Impact Factor (which is published in June 2014) fell to 4.72 from 5.11 for 2012 (as published in June 2013). The 2013 impact factor means that the JCMR papers that were published in 2011 and 2012 were cited on average 4.72 times in 2013. The impact factor undergoes natural variation according to citation rates of papers in the 2 years following publication, and is significantly influenced by highly cited papers such as official reports. However, the progress of the journal’s impact over the last 5 years has been impressive. Our acceptance rate is <25 % and has been falling because the number of articles being submitted has been increasing. In accordance with Open-Access publishing, the JCMR articles go on-line as they are accepted with no collating of the articles into sections or special thematic issues. For this reason, the Editors have felt that it is useful once per calendar year to summarize the papers for the readership into broad areas of interest or theme, so that areas of interest can be reviewed in a single article in relation to each other and other recent JCMR articles. The papers are presented in broad themes and set in context with related literature and previously published JCMR papers to guide continuity of thought in the journal. We hope that you find the open-access system increases wider reading and citation of your papers, and that you will continue to send your quality papers to JCMR for publication.

## Congenital heart disease, pulmonary arterial hypertension, pregnancy, and cardiovascular disease of the young

CMR of congenital heart disease is well established, although high quality acquisition and interpretation require training [1]. CMR is used in fetuses, [2, 3] children, [4] and adults, [5, 6] often as a complement to echocardiography, to avoid imaging with ionising radiation, and reduce the need for invasive diagnostic cardiac catheterisation [7]. CMR has a proven role in idiopathic pulmonary arterial hypertension and its potential utility in other forms of pulmonary arterial hypertension is under study. Three dimensional data acquisition is usual. 3D and 4D flow imaging, which allows the visualisation of large scale vorticity and the retrospective measurement of flow in vessels within the volume covered, continue to be of interest.

### Evaluation of a comprehensive cardiovascular magnetic resonance protocol in young adults late after the arterial switch operation for d-transposition of the great arteries

In this observational study, 27 adults with prior arterial switch operation (ASO) for transposition of the great arteries (D-TGA) underwent CMR for biventricular systolic function, myocardial scar burden, coronary ostial assessment and myocardial perfusion during vasodilator stress [8]. CMR provided conclusive diagnostic imaging for coronary ostia in most patients (24/27). CMR stress perfusion was normal in all. By contrast same day single photon emission computed tomography (institutional practice) imaging showed abnormalities in 54 % of cases; abnormalities were incongruent with anatomic coronary imaging and therefore considered false positives. Asymptomatic, clinically stable adult ASO patients have low pre-test probability for inducible ischemia suggesting routine surveillance with CMR stress perfusion is unnecessary.

### Cardiovascular magnetic resonance parameters associated with early transplant-free survival in children with small left hearts following conversion from a univentricular to biventricular circulation

This retrospective analysis reported CMR parameters associated with transplant free survival with a biventricular circulation after conversion of a univentricular circulation to a biventricular circulation in two patient groups [9]. In borderline hypoplastic left heart (*n* = 22), higher LV end diastolic volume (minimum 22 mL/m^2^), higher LV to RV stroke volume ratio (minimum 0.19) and higher mitral to tricuspid inflow ratio were associated with successful biventricular conversions. In unbalanced atrioventricular septal defect with right ventricular dominance (*n* = 10), which was a smaller subgroup with only one death, no association of CMR parameters with transplant-free survival with a biventricular circulation were found. Despite that this is a difficult topic to study with heterogeneous patients, and small sample size these data suggest that CMR parameters can be used to predict likelihood of successful biventricular repair in children with hypoplastic left heart. CMR predictors were superior to conventional echocardiographic measures.

### Cardiovascular magnetic resonance of cardiac function and myocardial mass in preterm infants: a preliminary study of the impact of patent ductus arteriosus

The authors studied 45 preterm infants of which 16 had a patent ductus arteriosus (PDA) [10]. EDV, SV, LV output, LV mass (corrected by weight) and EF decreased with increasing corrected gestational age in 29“healthy” neonates without a PDA. In the PDA group, shunt volume by phase contrast CMR correlated with increased LV mass corrected for postnatal age and gestational age and there was increased LV size and output compared with the non PDA group. However, ejection fraction and wall thickness were not different between preterm infants with a PDA and infants without a PDA suggesting that LV function is relatively maintained.

### Comparison of conventional autopsy and magnetic resonance imaging in determining the cause of sudden death in the young

Seventeen young patients with sudden death (mean age 23 ± 11 years, 71 % male) who died between 2010 and 2012 were studied with 1.5 T MR and CT prior to autopsy [11]. The most common cause of death was cardiac (47 %) including ARVC (24 %) and ischemic heart disease (12 %). MR diagnosis was concordant with autopsy in 12 patients, while in five cases, cause of death remained unknown despite autopsy. Dedicated post mortem MR imaging of the heart and brain may allow identification of cause of sudden death where conventional autopsy cannot be performed for logistical, cultural or personal reasons.

### Myocardial fibrosis in Eisenmenger syndrome: a descriptive cohort study exploring associations of late gadolinium enhancement with clinical status and survival

This group performed CMR with LGE in a cohort of 30 patients with Eisenmenger syndrome, mean age 43 ± 13 years, 20 female followed up for a median 7 years [12]. LGE was present in 73 % of patients, specifically in RV myocardium (70 %), RV trabeculae (60 %), LV myocardium (33 %) or LV papillary muscles (30 %). Myocardial fibrosis by LGE is common in Eisenmenger syndrome, though not extensive and in this pilot study its presence and quantity did not correlate with ventricular size, function, degree of cyanosis, exercise capacity, or survival in this pilot study suggesting that routine clinical use of LGE in these patients is not to date justified.

### Cardiovascular magnetic resonance in pregnancy: Insights from the cardiac hemodynamic imaging and remodeling in pregnancy (CHIRP) study

There is relative paucity of data related to expected haemodynamic changes in pregnancy assessed by CMR. The objective of the Cardiac Hemodynamic Imaging and Remodeling in Pregnancy (CHIRP) study was to compare transthoracic echocardiogram and CMR in the non-invasive assessment of maternal cardiac remodeling during the peripartum period [13]. Thirty four healthy pregnant women were recruited and studied during the 3rd trimester and at least 3 months post-partum (control values for the non-pregnant state). CMR reference values for the increase in LVEDV and LV mass associated with normal pregnancy are reported. Transthoracic echocardiography consistently underestimated LV volumes and mass compared to CMR. A limitation in this study is that postpartum 3 month findings are assumed equivalent to findings prior to pregnancy.

### The distribution and prognosis of anomalous coronary arteries identified by cardiovascular magnetic resonance: 15 year experience from two tertiary centres

This dual tertiary centre study retrospectively identified consecutive patients with an anomalous coronary artery originating from the opposite sinus of Valsalva (ACAOS, *n* = 116), of which 64 (55 %) had an inter-arterial course, passing between the aorta and pulmonary artery [14]. ACAOS patients had 58 MACE events at median 4.3 years follow up (five cardiovascular deaths, 5 PCI, 24 CABG and 24 myocardial infarctions). Events were significantly more common in patients with ACAOS and an inter-arterial course compared to those without an inter-arterial course, driven by surgical revascularization and myocardial infarction.

### Cardiovascular magnetic resonance assessment of ventricular function and myocardial scarring before and early after repair of anomalous left coronary artery from the pulmonary artery

This case series reports three Tesla CMR findings in eight patients with anomalous left coronary artery from the pulmonary artery (ALCAPA) before and early after (mean 4.9 ± 2.5 months) coronary reimplantation surgery [15]. The group studied eight ALCAPA patients prior to and early after coronary reimplantation. LV dilatation and dysfunction was present in all patients and improved significantly after surgery. Despite impaired myocardial perfusion and severely compromised LV function, only 2/8 patients had LGE enhancement pre-procedure which was associated with delayed recovery of LV function in one and was still present postoperatively in both. New onset transmural scarring occurred in two patients and in one left coronary artery stenosis was detected and required reoperation.

### High-resolution 3-dimensional late gadolinium enhancement scar imaging in surgically corrected Tetralogy of Fallot: clinical feasibility of volumetric quantification and visualization

LGE enhancement is associated with adverse clinical features in repaired tetralogy of Fallot (ToF) and RV scar is the substrate for ventricular arrhythmia. This study demonstrated high resolution 3D LGE at three Tesla findings in 15 patients using a whole heart, respiratory navigated technique and evaluated a semi-automated segmentation analysis [16]. Total right ventricular outflow tract scar volume using the semi-automated method was compared to manual segmentation as the gold standard in 15 patients with ToF. 14/15 patients had 3D imaging of sufficient quality for LGE quantification and this showed excellent agreement with manual segmentation with a significantly faster analysis time.

### Right ventricular adaptation to pulmonary pressure load in patients with chronic thromboembolic pulmonary hypertension before and after successful pulmonary endarterectomy - a cardiovascular magnetic resonance study

This is an insightful study of right ventricular adaptation to pressure loading conditions in 65 patients with chronic thromboembolic hypertension before and after pulmonary endarterectomy using CMR combined with haemodynamic measurements available from routine right heart catheterization [17]. The authors demonstrated that RV function is largely determined by afterload and returns to normal once ventriculo-arterial coupling and effective pulmonary arterial elastance are restored by pulmonary endarterectomy.

## Technical advances

The march of technological improvement never stops and the cardiovascular system inspires considerable activity, probably because it is challenging and ripe for continued development. Advances reported in the journal have included real-time imaging, [18] dual transmit RF, [19] and rapid functional assessment [20]. The full breadth of new developments is described in the papers in the section.

### Analysis of temperature dependence of background phase errors in phase-contrast cardiovascular magnetic resonance

This paper looks more deeply into the underlying issues behind the background phase errors that still represent a major problem with regard to the accuracy of clinical blood flow measurement [21]. The results showed the close relationship between gradient mount temperature changes during scanning and first order background phase shifts. The conclusion of this study is that advanced correction methods may well be required that take into account the gradient temperature and/or using of concurrent field-monitoring to map gradient-induced fields throughout the scan.

### Method for calculating confidence intervals for phase contrast flow measurements

This work was also focused on the subject area of the accuracy of phase contrast flow measurement but in this case on confidence intervals and how these were affected by noise associated with accelerated imaging [22]. The authors presented and validated a novel method for determining the standard deviation of flow or flow ratio measurements based on thermal noise in Cartesian retro-gated phase contrast CMR. Validation was achieved by comparison with repeated measurements in a phantom and pseudo replica reconstructions of in vivo data. The method could provide a useful tool directly integrated into flow analysis software packages where confidence intervals could be evaluated on the fly.

### Towards highly accelerated Cartesian time-resolved 3D flow cardiovascular magnetic resonance in the clinical setting

3D phase contrast CMR has traditionally been limited by long acquisition times. This study tested an 8-fold acceleration of Cartesian time-resolved 3D flow [23]. Data acquisition was feasible in both healthy controls and patients with complex congenital heart disease with a nominal scan time of 6–7 min. The 3D dataset allowed demonstration of circular and helical flow patterns within the Fontan circulation and showed good agreement with quantification performed using 2D flow velocity mapping.

### Dual echo positive contrast bSSFP for real-time visualization of passive devices during magnetic resonance guided cardiovascular catheterization

This innovative sequence development for real-time interventional CMR uses the concept of positive contrast of paramagnetic materials obtained with dephasing gradients in a dual echo sequence that allows both the dephasing gradient image and a more standard bSSFP image to be acquired simultaneously at frame rates of 5–6 frames/s [24]. The approach was demonstrated both in a phantom and a swine model of left heart cardiac catheterisation to visualise a nitinol guide wire. A near real-time image processing pipeline was used to display the guide-wire as an overlay on the bSSFP image during the procedure. A variable flip angle scheme was also used to reduce the specific absorption rate and hence device heating.

### T1-mapping in the heart: accuracy and precision

This state of the art review of T1 mapping discusses current methods for T1 quantification and provides comparison of the differing techniques for native and post-gadolinium measurement [25]. Both T1 values and extracellular volume (ECV) fraction have been shown to have prognostic value in a number of myocardial pathologies. Inversion recovery techniques such as MOLLI have excellent reproducibility when tightly controlled protocols are employed, although are susceptible to magnetization transfer. While saturation recovery methods may allow greater accuracy, they are more prone to artefact and currently less reproducible.

### Optimized saturation recovery protocols for T1-mapping in the heart: influence of sampling strategies on precision

In recent years there has been considerable interest in T1 mapping which has led to the development of a wide variety of methodology for measurement [26]. This paper represents a much needed comparison and parameter optimization of the saturation-recovery cardiac T1 mapping sequence. This includes a very detailed and rigorous evaluation of the 2 parameter SASHA sequence with variable flip angle for T1 mapping, pre and post Gd using theoretical computations, Monte Carlo simulations, and measurements from phantoms, and in vivo measurements in humans. The authors conclude that “the ability to quantify the measurement error has potential to determine the statistical significance of subtle abnormalities that arise due to diffuse disease processes involving fibrosis and/or edema and is useful both as a confidence metric for overall quality, and in optimization and comparison of imaging protocols”.

### Simultaneous three-dimensional myocardial T1 and T2 mapping in one breath hold with 3D-QALAS

The group presented a novel method of 3D interleaved T1 and T2 mapping which allowed acquisition of the entire left ventricular myocardium in a single breath hold [27]. When validated against MOLLI and Dual Echo measurements in-vivo, there was reasonable agreement for quantification of both T1 and T2 and excellent agreement for in-vitro images. This method allows rapid quantification of T1 and T2 in different tissues using CMR.

### MRXCAT: realistic numerical phantoms for cardiovascular magnetic resonance

With the massive potential in CMR, to implement differing sampling and reconstruction approaches there has been recent increased interest in simulators that can be used to help optimize sequence parameters and reduce artifacts for example [28]. This paper presents a novel framework for CMR simulation made available to the public via the internet. The focus is on sample characteristics (incl. motion), sampling and reconstruction issues rather than simulated acquisition accuracy. The so called MRXCAT is an extension for CMR of a previously developed simulation framework. In this study applications to cine and perfusion CMR are presented although extensions to other CMR modalities, such as diffusion or flow encoded MRI, are potentially possible.

### High performance MRI simulations of motion on multi-GPU systems

Another example of a CMR simulator again made available online is this simulator using a high performance multi-GPU environment [29]. The authors describe application of three different motion models that can be applied with this simulator, allowing simulation of large 3D models. As the authors explain, the incorporation of realistic motion models, such as heart motion and flow models may benefit the design and optimization of existing or new MR pulse sequences, protocols and algorithms that examine motion related MR applications. The computational speed enabled by the GPU for this is impressive.

### Quantitative three-dimensional cardiovascular magnetic resonance myocardial perfusion imaging in systole and diastole

In this paper the authors have performed a prospective study on 40 consecutive CAD patients to investigate the feasibility of a 3D perfusion CMR technique for detection of significant coronary disease [30]. The novel CMR sequence used k-t principal component analysis (PCA) reconstruction to enable a ten fold acceleration of the acquisition which in turn enabled two complete 3D images to be acquired per cardiac cycle. Using this the authors have also looked at the effect of acquisition time point within the cardiac cycle to ascertain whether there is any significant difference in myocardial perfusion reserve as well as quantitative myocardial blood flow between systolic and diastolic acquisitions. The study demonstrated the feasibility of quantitative 3D-perfusion CMR. Additionally, the results showed that there are significant differences in systolic and diastolic MBF estimates with both cardiac phases providing comparable diagnostic yield but at different thresholds. The systolic images had fewer artifacts and higher image quality, suggesting that this phase may be preferred for future acquisition of 3D perfusion data.

### Three-dimensional balanced steady state free precession myocardial perfusion cardiovascular magnetic resonance at 3 T using dual-source parallel RF transmission: initial experience

During the last year there has been continued interest in the use of dual-source parallel RF transmission to improve CMR; in this case it has been applied to bSSFP myocardial perfusion scanning at 3 T [31]. The parallel transmission was used with B1 shimming to improve the B1 homogeneity and provide a reduction in “SAR hot spots” allowing reduced TR for the bSSFP acquisition. Multiple aspects are covered in the study including B1 inhomogeneity, SNR and CNR, comparison of k-t BLAST and k-t PCA in undersampled reconstruction, image quality and artifacts. The sequence comparisons are made on 25 volunteers and five patients. The authors conclude that “three-dimensional bSSFP myocardial perfusion CMR using local RF shimming with dual source parallel RF transmission at 3 T is feasible and improves signal characteristics compared with TGRE but that image artefact remains an important limitation of bSSFP perfusion imaging at 3 T”. It remains unclear whether the improvements presented are specific to particular scanner platforms or generally available as an overall improvement to the CMR community as a whole.

### Myocardial arterial spin labeling perfusion imaging with improved sensitivity

Arterial spin labelling represents an alternative non contrast approach to perfusion measurement that has been more widely applied outside the heart [32]. As with most sequences the application in the heart is complicated by motion. This paper describes the application of parallel imaging to speed up the acquisition window to reduce the apparent physiological noise in myocardial blood flow (MBF) quantification. By reducing this noise more temporally consistent measurements of MBF are provided and this improves the sensitivity of the measurement.

### Cardiac gating calibration by the septal scout for magnetic resonance coronary angiography

In order to acquire sharp motion free images of details in the heart such as coronary arteries or small late gadolinium enhanced regions, it is necessary to accurately define the patient specific diastasis period of the heart cycle [33]. This paper describes a method for the detection of the diastasis period by one-dimensional projection ‘septal scout’ imaging along the ventricular septum acquired at high temporal resolution (10 ms). The application in this work is to improve the selection of gating window for 3D coronary artery angiography. The authors compare this method with the method currently widely used for this, cine imaging. Qualitative and quantitative analysis of vessel sharpness using the septal scout demonstrated significant improvement over the cine method. Furthermore, the Septal Scout was shown to provide diastasis windows that are in close agreement with those provided by TDE measurements of septal motion.

### Semi-automated left ventricular segmentation based on a guide point model approach for 3D cine DENSE cardiovascular magnetic resonance

The DENSE method of measuring myocardial strain is becoming more established at least with the CMR clinical research community and the following two papers describe and test methods of simplifying ventricular segmentation for the strain analysis [34]. In this paper a semi-automated segmentation algorithm is presented for 3D cine DENSE. The method uses a finite element model to capture the volumetric epicardial and endocardial surfaces and propagate each surface through the cardiac cycle using the inherent displacement properties found in DENSE MR data. In this preliminary report of the method the time for the segmentation process is shown to be significantly reduced. The authors claim this work to be a significant step towards the automation of 3D cine DENSE data analysis.

### Simplified post processing of cine DENSE cardiovascular magnetic resonance for quantification of cardiac mechanics

Another approach to simplifying the analysis is described and evaluated in this paper [35]. Using their technique, contour tracing can be reduced to three contours per slice, instead of two contours per time frame and this reduces interaction time significantly. The authors evaluated the method on a large institutional dataset (197 cases) of human and mouse data and found that the difference between the proposed method and tracing every time frame to be smaller than the inter-observer difference. They concluded that the proposed method can therefore be used to reduce processing time without affecting results.

## Diffusion tensor imaging of the heart

Diffusion tensor imaging (DTI) gives information on the mean intravoxel alignments of tissue microstructures using diffusion weighted imaging. It is used in particular in the brain, where larger diffusion values occur along nerve fibres in comparison to across fibres. The technique can also be used with considerably increased difficulty (due to motion) for the heart, however most work has been done in animals, or in ex-vivo human hearts, because of the high difficulty of deriving DTI data from the moving heart. Recently a more robust imaging sequence for in-vivo human cardiac DTI has been described which makes human studies more approachable. Recent work has shown in-vivo reproducibility in humans in hypertrophic cardiomyopathy, [36, 37] and histological validation in animals.

### In vivo cardiovascular magnetic resonance diffusion tensor imaging shows evidence of abnormal myocardial laminar orientations and mobility in hypertrophic cardiomyopathy

Cardiac DTI was performed at 3 T in HCM and control subjects and studied at end-systole and late diastole [38]. In controls, the angle of the second eigenvector of diffusion (E2A) relative to the local wall tangent plane was larger in systole than diastole, consistent with the expected changes of laminar orientation in the healthy heart. HCM hearts appeared hypercontracted in systole, with raised E2A, and retained high systolic-like angulation in diastole, consistent with failure of ventricular relaxation.

### Probing dynamic myocardial microstructure with cardiac magnetic resonance diffusion tensor imaging

This editorial, [39] reviews diffusion tensor imaging (DTI) in hypertrophic cardiomyopathy from Ferreira et al. [38]. It describes the microstructure of the heart including the change in fibre orientation seen from the epicardial to the endocardial surface, with large scale orientation of myocytes seen ex-vivo by DTI. They describe the technical challenges of in-vivo cardiac DTI, including long acquisition times and the challenge of differentiation of diffusional and bulk motion in the beating heart compared to ex-vivo.

### Intercentre reproducibility of cardiac apparent diffusion coefficient and fractional anisotropy in healthy volunteers

The authors validated a stimulated-echo diffusion tensor imaging (DTI) sequence using phantoms and assessed its reproducibility between two CMR centres at 3 T [40]. The same ten healthy volunteers were scanned using the DT-CMR sequence at the two centres less than 7 days apart and the apparent diffusion coefficient (ADC) and fractional anisotropy (FA) were calculated in a single mid-ventricular, short axis slice. Systolic ADC, systolic FA and diastolic FA were similar and only the diastolic ADC showed a statistically significant, but numerically small, difference between centres. The intercentre, intrasubject coefficients of variance were: systolic ADC 7 %, FA 6 %; diastolic ADC 7 %, FA 3 %.

## Atheroma, vascular, aorta, valves

Beside its key role in research, CMR is most frequently used clinically for the carotid arteries where it can be used to characterize thrombus, [41] plaque components, [42] plaque vulnerability, atheroma burden, natural history of progression, and response to treatment. However, peripheral artery angiography, [43–45] and the role in investigation of pulmonary hypertension, [46] vasculitis and systemic hypertension, [47] is also making progress. This is an area of CMR where contrast agents, [48] and 3 T has had an impact [49, 50]. CMR is widely used for assessment of the aorta in both congenital, [51] genetic, [52] and acquired conditions and is particularly well suited longitudinal follow-up of aortic dimensions, [53] and more complex aspects of aortic function, [54] such as pulse wave velocity, [55] distensibility and shear stress [56]. The design of arterial phantoms has proved useful for modelling [57]. The application of CMR to the assessment of valvular heart disease continues to increase, [58] particularly in aortic stenosis [59–61]. This is in part due to greater appreciation of its complementary roles in relation to echocardiography, which is commonly the first line investigative technique.

### Feasibility study of electrocardiographic and respiratory gated, gadolinium enhanced magnetic resonance angiography of pulmonary veins and the impact of heart rate and rhythm on study quality

Three dimensional respiratory and end systolic ECG gated, gadolinium enhanced magnetic resonance angiography was performed on a 3 Tesla (3 T) scanner in 101 consecutive patients, 35 of them in AF, prior to ablation [62]. Image and segmentation quality were scored. All studies except one (99 %) were considered diagnostic, 91 of them (90.1 %) being of good or excellent quality. Quality was not found to depend on heart rate or rhythm for this free breathing, radiation free strategy, which offers an alternative to current MRA or CT based approaches.

### Accelerated free breathing ECG triggered contrast enhanced pulmonary vein magnetic resonance angiography using compressed sensing

This is a feasibility study assessing an accelerated electrocardiogram-triggered contrast enhanced pulmonary vein MRA with isotropic spatial resolution, compressed sensing and respiratory navigator to define pulmonary venous anatomy prior PV ablation for atrial fibrillation in 19 patients and compared with the conventional breath-hold first pass non-ECG triggered CE-MRA [63]. The authors showed that the new technique provides an improved PVs sharpness compared to conventional technique and may be a considered as an alternative in patients in which the first pass CE-PV MRA fails due to inaccurate first pass timing or inability of the patient to perform a 20–25 s breath-hold. Important limitations of this study includes: small patient population, signal to noise ratio and contrast to noise ratio were not assessed and the benefit of the proposed approach for the planning and guidance of PVI procedures as well as the detection of potential post-procedural complications was not evaluated.

### Computed tomography angiography vs 3 T black-blood cardiovascular magnetic resonance for identification of symptomatic carotid plaques

This was a head-to-head comparison between Multi-detector Computed Tomography Angiography (MDCTA) and black-blood 3 T-cardiovascular magnetic resonance (bb-CMR) with respect to their ability to identify symptomatic carotid plaques characteristics [64]. Twenty-two stroke unit patients with unilateral symptomatic carotid disease and >50 % stenosis by duplex ultrasound were studied within 15 days of symptom onset. Both symptomatic and contralateral asymptomatic sides were evaluated. Bb-CMR was shown to be superior to MDCTA at identifying symptomatic carotid plaques, while MDCTA offers high specificity at the cost of low sensitivity. Results were only slightly improved over bb-CMR alone when combining both techniques. The study design, however, is cross sectional examining patients symptomatic patients and does not assess plaque characteristics regarding the risk of future cerebrovascular events. In addition the MDCTA examination parameters were not identical and this may have accounted for possible differences by applying a uniform image quality score to all images. Other limitation is contrast enhanced MRA was not used which might have increased the sensitivity of CMR to detect surface irregularities in carotid plaques.

### High resolution 3D diffusion cardiovascular magnetic resonance of carotid vessel wall to detect lipid core without contrast media

This mainly technical work aims to develop a diffusion-prepared turbo-spin-echo (DP-TSE) technique for carotid plaque characterization with 3D high resolution and improved image quality and applied to 15 healthy volunteers and six patients with carotid artery disease [65]. In this preliminary study the authors diffusion-prepared CMR allows 3D DWI of the carotid arterial wall in vivo with high spatial resolution and improved image quality. It can potentially detect lipid rich necrotic core without the use of contrast agents.

### HIV is an independent predictor of aortic stiffness

Cardiovascular events and cardiovascular related mortality increased in HIV-infected patients treated with effective antiretroviral therapy [66]. Identifying those at higher cardiovascular risk is therefore of great clinical importance. This study showed that, after matching for potential confounders, HIV infection is independently associated with increased aortic PWV and decreased aortic distensibility measured by CMR, both sensitive markers of reduced aortic elastic function. In addition HIV infection is an independent predictor of both increased pulse wave velocity and decreased aortic distensibility, clinical measures of aortic stiffness linked to increased cardiovascular mortality. The size of this detrimental effect is similar to that seen with the metabolic syndrome, a powerful cardiovascular risk factor. The authors showed that HIV and the metabolic syndrome are additive in their negative effects on PWV and aortic distensibility, suggesting that both are risk factors that act in different ways to impair vascular elasticity. The mechanism of vascular alterations in patients with HIV may be secondary to direct effects of the HIV virus on vascular function, including direct alteration in endothelial function, inflammation, and modification of aortic wall vascular smooth muscle cell behaviour and extra-cellular matrix composition. Due to the observational nature of this study, it is not possible to confirm causality or mechanisms which might underlie the increased aortic PWV and decreased aortic distensibility in patients with HIV. The study was also not powered to detect differences in HAART-naïve and treated subjects. Nor was it possible to determine the effects of individual anti-retroviral medications on vascular function.

### Optimization of Improved Motion-sensitized Driven-equilibrium (iMSDE) blood suppression for carotid artery wall imaging

Authors investigate the signal to noise ratio (SNR) and blood suppression performance of improved motion-sensitized driven-equilibrium (iMSDE) preparations technique for carotid wall imaging using composite RF pulses and sinusoidal gradients [67]. Twelve healthy volunteers and six patients with carotid artery disease underwent iMSDE and double inversion recovery (DIR) prepared T1- and T2-w fast spin echo (FSE) of the carotid arteries. iMSDE preparation achieved better blood suppression than DIR preparation with reduced vessel wall CNR efficiency in T1-w and T2-w images. However, SNR is significantly reduced and the patients population studied is extremely small.

### Scan-rescan reproducibility of quantitative assessment of inflammatory carotid atherosclerotic plaque using dynamic contrast-enhanced 3 T CMR in a multi-center study

Atheromatous plaques neovascularization and inflammation are associated with clinical cardiovascular events. Detection and quantification of neovascularization and inflammation in-vivo are important. This study aims to investigate the inter-scan reproducibility of kinetic parameters in atherosclerotic plaque using dynamic contrast-enhanced (DCE) CMR in a multi-center setting using 3 T field strength [68]. Study result indicates that quantitative measurement from DCE-CMR is feasible to detect changes with a relatively modest sample size in a prospective multi-center study despite the limitations. The relative high dropout rate suggested the critical needs for intensive operator training, optimized imaging protocol, and strict quality control in future studies.

### Multi-contrast atherosclerosis characterization (MATCH) of carotid plaque with a single 5-min scan: technical development and clinical feasibility

A 3D CMR technique using Multi-contrast Atherosclerosis Characterization (MATCH) was developed to address some aspects of CMR limitation in this field including low slice resolution, long scan time, image mis-registration, and complex image interpretation [69]. The advantage of MATCH is it provides spatially co-registered multi-contrast image sets in a single scan for characterization of carotid plaque composition. As the authors acknowledge there are several important limitations of this study including no provision of plaque histology and the extremely limited patient sample size, six patients only.

### Cardiovascular outcome associations among cardiovascular magnetic resonance measures of arterial stiffness: the Dallas heart study

The purpose of this study was to evaluate associations of CMR measures of total arterial compliance and two CMR measures of aortic stiffness with respect to future cardiovascular events in 2122 Dallas Heart Study participants without cardiovascular disease [70]. Study results showed that total arterial compliance and aortic distensibility may be stronger predictors of nonfatal cardiac events, while pulse wave velocity may be a stronger predictor of nonfatal extra-cardiac vascular events. Important limitations of this study include: non-invasive measurement of peripheral blood pressure to calculate aortic compliance and distensibility and the limited number of adverse events throughout the surveillance period limited the statistical power to explore associations with event subtypes and within subgroups.

### Robust volume-targeted balanced steady-state free-precession coronary magnetic resonance angiography in a breathhold at 3.0 Tesla: a reproducibility study

The purpose of this work was to refine, implement, and test a robust, practical single-breathhold bSSFP coronary MRA sequence at 3.0 T and to test the reproducibility of the technique [71]. Results showed that the 3D bSSFP acquisition, using a state-of-the-art MR scanner equipped with recently available technologies such as multi-transmit, 32-channel cardiac coil, and localized B0 and B1+ shimming, allows accelerated and reproducible multi-segment assessment of the major coronary arteries at 3 T in a single breathhold. The study is limited by small patient population (three patients with coronary artery disease and 15 healthy volunteers) and the imaging sequence was not evaluated in both the right and left coronary systems in every volunteer.

### Imaging of carotid artery vessel wall edema using T2-weighted cardiovascular magnetic resonance

The ability of novel 3D T2W black-blood imaging sequence to detect oedema induced in porcine carotid arteries by acute balloon injury compared to conventional 2D T2-weighted black-blood CMR was investigated in a porcine model [72]. The novel 3D imaging sequences and T2prep-GE perform comparably to conventional 2D T2-STIR in terms of detecting vessel wall edema. The improved spatial coverage of these 3D sequences may facilitate visualization of vessel wall edema to enable detection and monitoring of vulnerable carotid atherosclerotic plaques. A valid critique is that the balloon injury may cause more edema than there will be present in an atherosclerotic plaque although even very subtle injury to the vessel caused by pulling back the deflated balloon was reliably detected by CMR in accordance with macroscopical uptake by means of Evans Blue dye that only enters the injured vessel wall.

### Observational study of regional aortic size referenced to body size: production of a cardiovascular magnetic resonance nomogram

CMR is regarded the standard of reference for clinical assessment of the aorta, but normal CMR dimensions in large size population study are not available. The aim of this study is to produce a normal CMR reference range of aortic diameters and to investigate the relationship between regional aortic size and body surface area (BSA) in a large group of healthy subjects with no vascular risk factors [73]. A normal CMR reference range for BSA normalized aortic diameters, correlated to age at six points along the aorta were presented. Across both genders, increasing body size is characterized by a modest degree of aortic dilatation, even in the absence of traditional cardiovascular risk factors.

### Feasibility of asymmetric stretch assessment in the ascending aortic wall with DENSE cardiovascular magnetic resonance

The objective of this feasibility study is to investigate the feasibility of assessing asymmetric stretch in healthy and diseased ascending aortas using Displacement Encoding with Stimulated Echoes (DENSE) [74]. Fifteen patients with congenital aortic valve disease or aortic dilation and five healthy volunteers were studied. The study showed that evaluation of asymmetric stretch is feasible in the ascending aorta with DENSE CMR. Clear differences in stretch are seen between patients and volunteers, with asymmetric patterns demonstrated around the aortic circumference.

### Non-invasive imaging of carotid arterial restenosis using 3 T cardiovascular magnetic resonance

This study investigated the ability of 3 T CMR to determine the components of recurrent carotid artery disease and examined whether these differed from primary atherosclerotic plaque [75]. Twenty-five patients with previous carotid endarterectomy and 25 with primary asymptomatic atherosclerotic plaques serving as controls were studied. Two experienced reviewers analysed the multicontrast CMR images according to the presence or absence of major plaque features and assigned an overall classification type. As defined by CMR, restenotic lesions of the carotid artery fall into three distinct types and differ in composition from primary atherosclerotic plaques. If validated by subsequent histological studies, these findings could suggest a role for CMR in detecting high-risk (i.e. lipid-rich) restenotic lesions.

### The role of cardiovascular magnetic resonance in stratifying paravalvular leak severity after transcatheter aortic valve replacement: an observational outcome study

Paravalular leak (PVL) is common post transcatheter aortic valve implantation (TAVI). A population of patients post TAVI underwent CMR and echocardiographic assessment of the aortic valve for PVL [76]. Severity of PVL was greater when quantified using CMR compared to echocardiography. On follow up, 48 % of patients reached the primary endpoint of all cause death, heart failure hospitalisation and intractable symptoms requiring repeat invasive therapy. PVL with a regurgitant fraction >20 % was associated with reduced event free survival and CMR provided superior prognostic value compared to both quantitative and semi-quantitative echocardiographic assessment.

## Feature tracking

Measuring cardiac function is a fundamental for CMR. Myocardial tagging remains a source of research, [77] and at 3 T has attracted interest, partly because of the improved tag persistence with longer T1. Newer techniques have also been reported in humans, including myocardial velocity encoding, [78–80] and DENSE [81, 82]. Feature tracking has become an area of active interest with development of simple to use software that can be used post-hoc on simple cines [83, 84]. The papers below pursue novel aspects of cardiac function.

### Feature tracking compared with tissue tagging measurements of segmental strain by cardiovascular magnetic resonance

In this study, the CMR cine images of ten healthy volunteers, ten patients with left bundle branch block and ten with hypertrophic cardiomyopathy, who had also had CMR tissue tagging performed, were analysed by feature tracking (FT) of both the endocardial and mid wall circumferences in short axis planes [85]. The intra and inter-observer agreements of segmental peak systolic circumferential strain and time to peak systolic circumferential strain were substantially lower for feature tracking than tagging, leading to the conclusion that, although convenient, the feature tracking techniques used were less suitable than the tagging for clinical or research purposes.

### Biventricular myocardial strain analysis in patients with arrhythmogenic right ventricular cardiomyopathy (ARVC) using cardiovascular magnetic resonance feature tracking

This study aimed to examine whether CMR based strain analysis using feature tracking (FT) could serve as a quantifiable measure to confirm global and regional ventricular dysfunction in ARVC patients and so support the early detection of ARVC [86]. Groups studied were 20 with ARVC, 30 with borderline ARVC, 22 with a positive family history but no manifest ARVC and ten healthy volunteers. Strain analysis by FT was found to differentiate between manifest or borderline ARVC and the healthy volunteers HV, even when ejection fraction was normal.

### Quantification of left atrial strain and strain rate using Cardiovascular Magnetic Resonance myocardial feature tracking: a feasibility study

Myocardial feature tracking (FT) was applied to the SSFP long axis cine images of ten healthy volunteers, ten patients with hypertrophic cardiomyopathy (HCM) and ten with heart failure and preserved ejection fraction (HFpEF) to quantify left atrial deformation [87]. Evidence of impaired LA reservoir function was found in HCM and HFpEF relative to that of volunteers suggesting that this novel approach has potential value for the quantification of atrial performance.

### Value of additional strain analysis with feature tracking in dobutamine stress cardiovascular magnetic resonance for detecting coronary artery disease

This study assessed the value of feature tracking for assessment of myocardial ischaemia [88]. Circumferential strain of the LV (Fcc) was measured during Dobutamine stress CMR (DS-CMR) in a population of 25 patients with known or suspected coronary artery disease and normal resting left ventricular function. There was no difference in Fcc between myocardial segments supplied by unobstructed coronary arteries (defined angiographically) and those supplied by significantly stenosed arteries at rest, however there was a significant difference between the two groups following Dobutamine infusion. Using angiography as the gold standard, Fcc during Dobutamine differentiated normal from stenotic segments with a sensitivity of 75 %, a specificity of 67 % and an area under the curve of 0.78.

## Cardiomyopathy

The phenotyping of cardiomyopathy is a primary clinical indication for CMR, and has become mainstream in hypertrophic cardiomyopathy. Recent attention has been focussed on T1 imaging, [89, 90] with standardisation of T1 acquisition, [91–93] technical developments, [94–96] and the assessment of diffuse fibrosis in cardiomyopathy [97]. Significant progress has been made in a wide range of unusual conditions including muscular dystrophy, [98–100] ARVC, [101] systemic sclerosis, [102] cardiac related cardiac dysfunction, [103] non-compaction, [104, 105] and iron loading [106–109].

### Prevalence of myocardial crypts in a large retrospective cohort study by cardiovascular magnetic resonance

Recent reports suggest that crypts, meaning discrete clefts or fissures in the myocardium of the left ventricle (LV), usually inferobasal, are prevalent in phenotypically positive or negative carriers of genes associated with hypertrophic cardiomyopathy (HCM). However, the retrospective study by Child et al. of 1020 consecutive patients or volunteers who had undergone CMR during a 12-month period also identified crypts in healthy controls (11/306, 3.6 %), there being crypts identified in 64 (6.3 %) of all of the 1020 CMR studies [110]. Crypts were more prevalent in non-ischaemic cardiomyopathy subgroups (9/76, 11.7 %, in patients with HCM and 3/11, 27 %, in those with hypertensive cardiomyopathy). They were also more prevalent in those referred for CMR for family screening of inherited forms of cardiomyopathy (10/41, 23 %), although the prognostic significance of crypts in such individuals remains uncertain until prospective studies of sufficient numbers have been performed and analysed.

### Population-based studies of myocardial hypertrophy: high resolution cardiovascular magnetic resonance atlases improve statistical power

In this study, LV short-axis cine images were acquired in 138 healthy volunteers using standard 2D imaging and 3D high spatial resolution CMR [111]. A multi-atlas technique was used to segment and co-register each image. High spatial resolution CMR with automated phenotyping provides greater power for mapping wall thickness than conventional 2D imaging, enabling a reduction in the sample size required for studies of environmental and genetic determinants of LV wall thickness.

### Reproducibility of native myocardial T1 mapping in the assessment of Fabry disease and its role in early detection of cardiac involvement by cardiovascular magnetic resonance

Native T1 mapping was assessed using MOLLI and ShMOLLI sequences at 1.5 T in 63 patients with genetically-confirmed Fabry disease, 25 of whom did not have left ventricular hypertrophy (LVH) [112]. Native T1 was lowest in the cohort of Fabry’s with LVH, then Fabry’s without LVH then the control population. The technique showed high inter- and intra-observer agreement. Reduced native myocardial T1 was associated with reduced global longitudinal strain on echocardiographic speckle tracking and increased E/E’, indicating it may underlie early diastolic dysfunction in Fabry’s.

### Reference values for healthy human myocardium using a T1 mapping methodology: results from the International T1 Multicenter cardiovascular magnetic resonance study

This trial determined the reference range for T1 mapping through study of healthy controls and low risk subjects using a standardized sequence (MOLLI, scheme 3(3)3(3)5)) on 1.5 and 3 T Phillips platforms across multiple hospital sites [113]. There was close agreement between T1 values obtained in the core lab and locally for all sites. Inter and intra operator reproducibility was excellent for native septal T1. Native T1 values appeared more reproducible than post-contrast T1 values.

### Accuracy and reproducibility of semi-automated late gadolinium enhancement quantification techniques in patients with hypertrophic cardiomyopathy

The group compared semi-automated LGE quantification techniques using manual segmentation as the reference standard in a cohort of 76 HCM patients [114]. Quantification was performed using full width half maximum (FWHM), signal threshold reference mean (STRM) and Otsu-auto-threshold (OAT). STRM > 3 standard deviations (SD) showed the greatest agreement with manual segmentation, however FWHM had the greatest inter-operator reproducibility. STRM > 5SD, 6SD and FWHM systematically underestimated total LGE volume compared to manual segmentation

### Cardiovascular magnetic resonance characterization of left ventricular non-compaction provides independent prognostic information in patients with incident heart failure or suspected cardiomyopathy

Patients with incident heart failure or suspected cardiomyopathy in whom CMR yielded a diagnosis of LV non compaction were followed for a mean of 27 months [115]. On multivariate analysis, the incidence of tachyarrhythmias (atrial and ventricular) was significantly greater in patients with LGE compared to those without LGE, even when adjusted for LV and RV ejection fraction.

### Left ventricular systolic function and the pattern of late-gadolinium-enhancement independently and additively predict adverse cardiac events in muscular dystrophy patients

Florian assessed 88 patients with Duchenne and Becker muscular dystrophies with CMR and followed them for a 47+/−18 months [116]. During this time there were three deaths, eight heart-failure hospitalisations and 13 episodes of non-sustained ventricular tachycardia. On multivariate analysis, LVEF and the presence of transmural LGE were the only independent predictors of hospitalization/non sustained ventricular tachycardia.

### Coronary microvascular ischemia in hypertrophic cardiomyopathy - a pixel-wise quantitative cardiovascular magnetic resonance perfusion study

The group used CMR first pass myocardial perfusion to perform pixel wise quantitative perfusion analysis in 35 HCM patients [117]. Myocardial blood flow was significantly higher in the LV endocardium compared to the epicardium at rest and this reversed following administration of adenosine. Segmental analysis revealed that 31 % of patients had stress myocardial perfusion values lower than rest perfusion, indicating regions of myocardial ischaemia. These were more common in regions with a greater wall thickness and more replacement fibrosis.

### Myocardial fibrosis in patients with myotonic dystrophy type 1: a cardiovascular magnetic resonance study

Myotonic dystrophy type 1 (DM1) is associated with increased cardiac morbidity and mortality. This CMR study of 30 DM1 patients revealed a high prevalence of myocardial fibrosis (40 %), most commonly of the RV insertion points and with a heterogenous distribution within the LV myocardium [118]. Presence of LGE was not predicted by ECG, Holter-monitoring or echocardiography. CMR for screening is additive to current standard cardiac assessment and may prove to be a clinically valuable tool for risk stratification in DM1.

### Patterns of late gadolinium enhancement in Duchenne muscular dystrophy carriers

Thirty carriers of the Duchenne muscular dystrophy (DMD) mutation were compared to gender and age matched controls [119]. A typical myocardial LGE-pattern location, localized to LV segments five and six was commonly seen in DMD carriers. LGE was more frequently subepicardial plus mid-myocardial in symptomatic carriers, despite normal LV systolic and diastolic function. No genotype-phenotype correlation was found.

### Effect of the 2010 task force criteria on reclassification of cardiovascular magnetic resonance criteria for arrhythmogenic right ventricular cardiomyopathy

Liu et al. evaluated the effect of the use of the revised 2010 Task Force Criteria for the diagnosis of ARVC through retrospective review of 968 consecutive patients referred for CMR with clinical suspicion of ARVC between 1995 and 2010 [120]. Application of the 2010 criteria resulted in reduction in the number of patients meeting CMR criteria for diagnosis from 22.7 to 2.6 % compared to the 1994 criteria.

### Arrhythmogenic right ventricular cardiomyopathy (ARVC): cardiovascular magnetic resonance update

This highly accessed Review article provides an update of Arrhythmogenic Right Ventricular Cardiomyopathy (ARVC), its diagnostic criteria, implicated genes, CMR evaluation and current management considerations [121]. ARVC is a common cause of sudden death in the young. CMR provides accurate measurement of RV volumes, a key criterion in the revised Task Force criteria as well as detailed characterization of cardiac morphology, function and tissue characterization. Pathogenic mutations in the cardiac desmosome are found in approximately 60 % of index patients and left ventricular involvement is increasingly recognized.

### A novel and practical cardiovascular magnetic resonance method to quantify mitral annular excursion and recoil applied to hypertrophic cardiomyopathy

This proof of concept paper described a novel CMR technique to evaluate mitral annual motion through tracking of the atrioventricular junction (AVJ) [122]. This was tested through retrospective analysis of 24 HCM and 14 healthy volunteers. The tracking feature allowed calculation of the maximum AVJ displacement during the cardiac cycle, the velocity of the mitral annulus during early diastole and in diastasis. HCM patients demonstrated significant reduction in mitral annual excursion compared to controls.

### In-vivo T1 cardiovascular magnetic resonance study of diffuse myocardial fibrosis in hypertrophic cardiomyopathy

Brouwer et al. compared T1 mapping derived ECV, acquired using a MOLLI sequence, in HCM and healthy controls [123]. Areas of LGE enhancement were excluded. There was no difference in ECV in HCM compared to controls for the regions of myocardium without LGE. The authors concluded that the additional clinical value of T1 mapping in HCM appeared limited.

### Radiation recall reaction causing cardiotoxicity

The authors describe the imaging findings of radiation recall reaction causing cardiotoxicity [124]. The patient had prior mediastinal radiotherapy and presented 5 months later with impaired ejection fraction after chemotherapy. CMR demonstrated regional myocardial edema on T2 weighted sequences and LGE in the same area.

### Subclinical myocardial inflammation and diffuse fibrosis are common in systemic sclerosis – a clinical study using myocardial T1-mapping and extracellular volume quantification

CMR was performed in 19 patients with systemic sclerosis and 20 controls at 1.5 T with cine imaging, tagging, T1-mapping, STIR, LGE imaging and ECV quantification [125]. Cardiac involvement was common, even in the absence of cardiac symptoms. 53 % of systemic sclerosis patients had LGE enhancement and focal areas of myocardial oedema on STIR imaging. The ECV was expanded, likely representing a combination of low-grade inflammation and diffuse myocardial fibrosis, and correlated with disease activity.

### Long term effects of cocaine on the heart assessed by cardiovascular magnetic resonance at 3 T

Ninety-four consecutive asymptomatic cocaine users with a mean duration of 14+/−9 years of drug use underwent CMR [126]. Compared to an age-matched healthy cohort, the cocaine abusers had increased LV end-systolic volume, LV mass index and RV end-systolic volume, with decreased LVEF and RVEF. No subject had myocardial oedema, but 30 % had myocardial LGE indicating myocardial damage. Duration of cocaine abuse was related to probability of LV systolic dysfunction.

### Mapping iron in human heart tissue with synchrotron x-ray fluorescence microscopy and cardiovascular magnetic resonance

This ex-vivo case study compared synchrotron X-ray fluorescence microscopy (XFM) elemental iron maps with magnetic resonance transverse relaxation rate maps of cardiac tissue samples from an iron-loaded patient with Diamond Blackfan anaemia [127]. This allowed comparison of macroscopic iron distribution within the heart with a high spatial resolution and comparison with regional T2* values using CMR. There was close qualitative and quantitative agreement between the synchrotron XFM iron maps and MR relaxometry maps. Iron appeared to preferentially load into the lateral epicardium wall and there was a strong gradient of decreasing iron, T2 and T2* relaxation properties from the epicardium to the endocardium.

### Calibration of myocardial T2 and T1 against iron concentration

Ex-vivo iron loaded hearts were studied using R1 and R2 measurements (R1 = 1/T1 and R2 = 1/T2) at 1.5 Tesla and compared with myocardial iron concentration calculated using inductively coupled plasma atomic emission spectroscopy [128]. There was a strong correlation between LnR2 and Ln[Fe] suggesting that T2 may provide additive information to T2* in myocardial siderosis. Ex-vivo T1 measurements were strongly affected by formalin and the authors conclude that T1 calibration may only be practical in-vivo or non-formalinised specimens.

### Biopsy-based calibration of T2* magnetic resonance for estimation of liver iron concentration and comparison with R2 Ferriscan

Liver iron concentration studies based on early T2* sequences were limited by a requirement for short echo times [129]. MR technology has since improved as has estimation of iron concentration on biopsy. The group performed a calibration study of a contemporary state of the start liver optimized T2* sequence using 50 iron-loaded liver biopsy specimens. There was a strong linear correlation between ln(T2*) and ln(LIC). The contemporary method yielded liver iron concentration values 2.2 times higher compared to the original proof of concept T2* values. Inter-observer agreement was excellent.

## Animal studies and MR spectroscopy

The use of animals to study cardiac physiology and metabolism continues to contribute a small but important section to the journal, often using MR spectroscopy, [130] and high field magnets, [131] and investigating novel ideas [132].

### Endogenous assessment of chronic myocardial infarction with T_1_ρ-mapping in patients

The longitudinal relaxation time in the rotating frame (T_1ρ_) is a tissue marker to non-invasively measure collagen content, and may have the potential to detect fibrosis in the hearts of patients with chronic myocardial infarction (MI) without the need of exogenous contrast agents [133]. This paper demonstrates the feasibility of native T_1ρ_-mapping for the detection of myocardial fibrosis in the clinical setting using a large animal model, and translates this technique into patients with chronic MI. While significantly higher T_1ρ_ –values were found in the infarct tissue of MI patients compared to the remote zone, the sensitivity of detecting the injury was lower compared to the gold-standard late gadolinium enhancement (LGE) CMR. This work opens a new avenue for non-invasive tissue characterization and further work is required to improve sensitivity and specificity of this technique.

### A high-resolution cardiovascular magnetic resonance diffusion tensor map from ex-vivo C57BL/6 murine hearts

The complex cardiac micro-structure and spatial arrangement of cardiomyocytes in laminar sheetlets is important for normal function of the heart. Cardiac diffusion tensor imaging (CDTI) of hearts *ex vivo* allows for a more detailed and comprehensive characterization of the myocardial architecture. In this study, high-resolution CDTI was combined with statistical tensor analysis to obtain an average description (i.e. atlas) of the fiber architecture in fixed mouse hearts [134]. The excellent spatial resolution and the high-signal-to-noise ratio of the data resulted in low variability of local myocyte orientation, leading also to the suggestion that hearts of mice are more structured than those of larger mammalians (including humans). This work represents a baseline of CDTI indices in mice, and may help to improve detection of biomarkers, particularly in disease states or following genetic modifications.

### Quantitative assessment of magnetic resonance derived myocardial perfusion measurements using advanced techniques: microsphere validation in an explanted pig heart system

This paper reports on the validation of quantitative cardiovascular Magnetic Resonance (CMR) myocardial perfusion imaging against fluorescently-labeled microspheres on an isolated perfused pig heart model in order to determine the accuracy of available algorithms for the quantification of myocardial blood flow (MBF) [135]. Perfusion images were acquired at 1.5 and 3 T at different perfusion states resembling physiological and reduced flow, during adenosine-induced hyperaemia and during coronary occlusion, respectively. The administration of the fluorescently-labeled microspheres and externally controlled coronary blood flow served as reference standards. It was found that the analysis based on the Fermi function deconvolution achieved the highest accuracy and best correlation at both field strengths, and may thus serve as the clinical tool providing accurate quantitative blood flow assessment.

### Myocardial perfusion and oxygenation are impaired during stress in severe aortic stenosis and correlate with impaired energetics and subclinical left ventricular dysfunction

A comprehensive, multi-parametric CMR protocol, including cine-MRI, tagging, BOLD, stress-perfusion and 31P-MRS was used to address the question whether or not reduced myocardial perfusion reserve observed in patients with left-ventricular hypertrophy due to aortic stenosis (AS) but without obstructive coronary artery disease leads to tissue deoxygenation [136]. This study found a blunted oxygenation response to adenosine stress in this patient group, which is suggestive of microvascular impairment. Both BOLD response and myocardial perfusion reserve index correlated with impaired energetics and subclinical LV dysfunction. Importantly, myocardial perfusion, oxygenation, energetics and contractility were restored following aortic valve replacement. This work nicely illustrates the strength of CMR in diagnosing and aiding novel therapeutic strategies in AS patients.

### Non-invasive assessment of cardiac function and pulmonary vascular resistance in a canine model of acute thromboembolic pulmonary hypertension using 4D flow cardiovascular magnetic resonance

This study sought to simultaneously quantify cardiac function and cardiopulmonary hemodynamic parameters from a free-breathing 4D (time-resolved three-directionally motion encoded) flow CMR examination in a canine model of acute thromboembolic pulmonary hypertension (PH) [137]. The accelerated 4D-technique allowed for the assessment of left- and right-ventricular functional indices in agreement with conventional 2D cine SSFP-derived values, while providing a comprehensive characterization of pulmonary artery and aortic flow, and tricuspid valve regurgitation velocity. However, this work demonstrates only feasibility in a small sample size, and does not validate the model in a separate cohort as acknowledged by the authors. Future work is required to investigate if these results are transferable to other etiologies of PH and/or humans.

### In vivo characterization of rodent cyclic myocardial perfusion variation at rest and during adenosine-induced stress using cine-ASL cardiovascular magnetic resonance

In this study, arterial spin labeling (ASL)-cine MRI was used to quantify variations in myocardial blood flow during a cardiac cycle in healthy rats [138]. The authors demonstrated a significant increase in MBF from end-systole to end-diastole in both rest and adenosine-induced stress conditions. These findings are in contrast to a previous study in humans, [139] which only reported differences in MBF under stress conditions but not at rest. Improved precision in the here presented small-animal study, which did not rely on a first-pass gadolinium-based MRI technique, may explain this discrepancy. The authors conclude that this technique may be useful to study microvascular defects in rodent models of non-ischemic heart disease.

### Chronic diuretic therapy attenuates renal BOLD magnetic resonance response to an acute furosemide stimulus

In this study, BOLD imaging was used in patients with renal artery stenosis (RAS) to characterize increases in renal oxygenation, as a marker of kidney viability, following the administration of a standard 20 mg intravenous furosemide stimulus [140]. In particular, it was shown that a chronic loop diuretic therapy attenuates the BOLD MR responses to an acute furosemide stimulus. This has significant implications for the evaluation of RAS patients being for renal artery revascularization procedures, suggesting that a different dosing strategy is required before BOLD MR can be widely used to determine renal viability.

## Left ventricular mass and function

Although it is possible to get diagnostic images in the majority of patients referred for CMR, image quality can be compromised by patient difficulty performing breath holds and also by arrhythmia. We have received a number of papers validating sequences aimed at overcoming difficulties, [141] and also reducing scan duration with benefit for both patients and workflow. Use of accelerated real-time data is one such approach to this problem, as it can reduce or eliminate the requirement for ECG gating and breathholding [142, 143]. There is also continued interest in novel analysis techniques, [144] novel areas, [145] reference ranges, [146] and atlas based studies for description of function for populations [147].

### Evaluation of left ventricular ejection fraction using through-time radial GRAPPA

In this paper, the authors determined LV volumes and LVEF using a free breathing real-time radial GRAPPA sequence at 1.5 T in 63 subjects comparing results to a standard breath-hold SSFP sequence [148]. The differences in EF, EDV, and ESV between the gold-standard and real-time methods were not statistically significant (*p*-values of 0.77, 0.82, and 0.97, respectively). Although there is a relatively lengthy calibration phase required for this technique, the scan time was significantly shorter for the highly accelerated real-time data collection (*p* < 0.001) and fewer artifacts were reported in the real-time images (*p* < 0.01). In the qualitative image analysis, reviewers marginally preferred the standard images although some features including cardiac motion were equivalently rated. The authors suggest that real-time functional CMR with through-time radial GRAPPA performed without ECG-gating under free-breathing can be considered as an alternative to gold-standard breathhold cine imaging for the evaluation of ejection fraction. As such, this can now be further evaluated in the more challenging groups to image.

### Quantification of left ventricular functional parameter values using 3D spiral bSSFP and through-time Non-Cartesian GRAPPA

The authors presented data on the initial validation of a novel 3D cine volumetric acquisition using multiple acceleration techniques [149]. A six-fold undersampled 3D stack of spirals balanced SSFP sequence with 3D through-time spiral GRAPPA parallel imaging reconstruction is compared to standard 2D cine imaging at 3 T for quantification of left ventricular EDV, ESV, EF and ventricular mass in 10 healthy volunteers. The 3D method demonstrated equivalent systolic left ventricular functional parameter values, required significantly less total scan time (48 s including a single 14 s breathhold compared to an average of 5 min 40 s for the same coverage by the 2D method) and yielded acceptable image quality with respect to the 2D segmented multi-breathhold standard.

### Ex vivo cardiovascular magnetic resonance measurements of right and left ventricular mass compared with direct mass measurement in excised hearts after transplantation: a first human SSFP comparison

This study has evaluated ex vivo LV and RV mass CMR assessment by SSFP sequence in comparison with autopsy mass of hearts from cardiac transplants patients in a single centre study of 55 explanted cardiomyopathy hearts which were successfully scanned just after surgery and then weighed by a pathologist [150]. Significant positive correlations were found between total 3D CMR mass (450 ± 111 g) and total pathology mass (445 ± 116 g; *r* = 0.99, *p* < 0.001) as well as 3D CMR measured LV mass (301 ± 93 g) and the pathology measured LV mass (313 ± 96 g; *r* = 0.95, *p* < 0.001). Strong positive correlations were demonstrated between the 3D CMR measured RV mass (149 ± 46 g) and the pathology measured RV mass (128 ± 40 g; *r* = 0.76, *p* < 0.001). The mean bias between 3D-CMR and pathology measures for total mass, LV mass and RV mass were: 3.0, −16 and 19 g, respectively. This paper validates the accuracy of SSFP-CMR sequence to determine myocardial mass as a ‘gold standard’ in humans.

### Left ventricular shape variation in asymptomatic populations: the multi-ethnic study of atherosclerosis

The authors successfully constructed an atlas comprising 1991 CMR cases contributed from the Multi-Ethnic Study of Atherosclerosis baseline examination. From this collection of data from asymptomatic adult volunteers, they quantified the dominant components of global shape variation in sub-cohorts (defined by ethnicity, sex, smoking, hypertension and diabetes) [151]. The data and results are available at cardiacatlas.org. After correction for height, shape distributions were principally explained by size, sphericity and concentricity, which are known correlates of adverse outcomes. The resulting shape components distinguished differences due to ethnicity and risk factors with greater statistical power than traditional mass and volume indices. This work is pertinent for the new generation of population based studies incorporating CMR, demonstrating the feasibility of such an analysis pipeline in large scale studies, and showing the incremental benefit of an atlas approach over standard analysis techniques.

### The influence of pericardial fat upon left ventricular function in obese females: evidence of a site-specific effect

This paper explores whether the previously reported association between increased pericardial fat volume and cardiac dysfunction is due to a systemic process or due to a direct local interaction. 60 obese females underwent CMR to assess LV function and pericardial fat volumes [152]. The authors found that LV fat correlated weakly with cardiac output (*r* = −0.41, *p* = 0.001) and stroke volume (*r* = −0.26, *p* = 0.05), as well as diastolic functional parameters including peak-early-filling rate (*r* = −0.38, *p* = 0.01) and early late filling ratio (*r* = −0.34, *p* = 0.03). LV hemodynamic and diastolic function was associated more with LV fat as compared to RV or total pericardial fat, but not with systemic inflammatory markers or adipokines. These findings suggest a site-specific influence of pericardial fat on LV function, which could imply local secretion of molecules into the underlying tissue or an anatomic effect.

## Diastolic function

The ability of echocardiography to rapidly and robustly evaluate diastolic function is one of its strengths. Although CMR techniques to reliably assess diastolic function have existed for many years, numerous methods for making this more routine within the CMR exam continue to materialise, [153, 154] often employing custom-written software and semi-automation. These techniques can vary from utilising tracking of anatomical features with time to more complex mathematical and physiological models.

### Evaluation of diastolic function by three-dimensional volume tracking of the mitral annulus with cardiovascular magnetic resonance: comparison with tissue Doppler imaging

In this study, the authors evaluate a semi-automated CMR technique for quantifying global LV diastolic function, using 3D volume tracking of the mitral annulus (MA) from conventional 2D cine-CMR images [155]. One hundred and twenty four consecutive patients with normal ejection fraction underwent both clinically indicated transthoracic echocardiography (TTE) and CMR. Interpolated 3D reconstruction of the MA over time was performed with semi-automated atrioventricular junction (AVJ) tracking in long-axis cine-SSFP images, producing an MA sweep volume over the cardiac cycle. Patients with TTE-based diastolic dysfunction (*n* = 62) showed significantly different normalized MA sweep volume profiles compared to those with TTE-based normal diastolic function. Good correlations were observed between CMR early-to-late mitral sweep rate (PSRE/PSRA) and early-to-late diastolic annular velocity ratios (e’/a’) measured by TDI (*r* = 0.756 to 0.828, *p* < 0.001). 3D MA sweep volumes generated by semi-automated AVJ tracking in routinely acquired CMR images yielded diastolic parameters that were effective in identifying patients with diastolic dysfunction when correlated with TTE-based variables.

### Left ventricular torsion shear angle volume analysis in patients with hypertension: a global approach for LV diastolic function

In this paper, the authors propose a novel index for evaluating LV diastolic function utilizing the LV normalized torsion shear angle volume loop [156]. They evaluated this in 60 resistant hypertension patients (HTN) and 40 control volunteers studied using CMR with tissue tagging. The area within the loop was termed the torsion hysteresis area and was found to be significantly increased (*p* < 0.001) in the hypertensive patients with concentric remodelling compared to controls and also correlated with E/A (*r* = 0.23, *P* = 0.025). The authors conclude that the normalised torsion shear angle volume loop incorporates both the active and passive recoil processes of LV diastolic and systolic phases, providing a new global description of LV diastolic function.

## Myocarditis

The use of CMR in the diagnosis and assessment of myocarditis has become commonplace with greater appreciation of the value of LGE and T2W oedema imaging in identifying this common but often challenging pathology, in addition to the merits of CMR for the assessment of ventricular remodelling.

### Cardiovascular magnetic resonance risk stratification in patients with clinically suspected myocarditis

In this paper, the authors assess the prognostic value of a normal CMR scan (defined by LV volumes and ejection fraction and the absence of LGE) in a single centre cohort of 405 consecutive patients with clinically suspected myocarditis followed up prospectively for median time of 1591 days [157]. CMR diagnosis was “myocarditis” in 28.8 %, “normal” in 55.6 % and “other pathology” in 15.6 %. The authors used composite endpoints for their analyses, while obtaining an overall mortality of 3.2 %. All (7) cardiac deaths and (3) events occurred in the group with abnormal CMR findings. Kaplan-Meier analysis showed significant difference for major adverse cardiac events (cardiac death, sudden cardiac death (SCD), ICD discharge, aborted SCD) between patients with normal and abnormal CMR (*p* = 0.0003). Based on their data, the authors conclude that patients with suspected myocarditis yet absence of LV dysfunction and absence of LGE images have a good prognosis independent of their clinical symptoms and other findings.

### Native T1-mapping detects the location, extent and patterns of acute myocarditis without the need for gadolinium contrast agents

T1 mapping continues to generate much additional data with a variety of sequences applied to a widened spectrum of myocardial pathologies in pursuit of identifying and demonstrating the areas where this technique has greatest clinical utility. In this paper, Ferreira et al. study 60 patients with suspected acute myocarditis and suggest that their technique based on native T1mapping (ShMOLLI) can reveal the typical non-ischemic regional patterns of disease, negating the need for Gadolinium contrast [158]. A threshold of T1 > 990 ms (sensitivity 90 %, specificity 88 %) detected significantly larger areas of involvement than conventional T2W and LGE imaging in patients, and additional areas of injury when T2W and LGE were negative. T1-mapping significantly improved the diagnostic confidence in an additional 30 % of cases when at least one of the conventional methods (T2W, LGE) failed to identify any areas of abnormality. Using incremental thresholds, T1-mapping can display the non-ischemic patterns of injury typical of myocarditis. Such data may lead to less routine requirement for the use of Gadolinium contrast.

## Cardiac resynchronisation therapy

The quest for enhanced predictors of response to cardiac resynchronisation therapy (CRT) and improved benefit from CRT implantation continues, with significant data assessing the role of CMR in this field.

### Relationship between mechanical dyssynchrony and intra-operative electrical delay times in patients undergoing cardiac resynchronization therapy

This study compares mechanical delay times derived from high temporal resolution short-axis cine CMR data with intra-operative electrograms recorded at multiple locations throughout the coronary sinus and its tributaries [159]. The authors found a strong correlation between electrical and mechanical delay times within each patient (R^2^ = 0.78 ± 0.23). They demonstrate that MRI can potentially be used to prospectively determine the ideal LV lead location, and show that within the coronary veins, the site of latest mechanical delay is the site of the latest electrical delay in the majority of patients (91 %). This study provides initial evidence that non-invasive mechanical activation patterns derived from CMR can accurately reflect the underlying electromechanical substrate of intraventricular dyssynchrony.

### A prospective evaluation of cardiovascular magnetic resonance measures of dyssynchrony in the prediction of response to cardiac resynchronization therapy

In this study, the authors establish and then evaluate a systolic dyssynchrony index (SDI) derived from CMR-based data as a predictor of response to CRT. SSFP and CSPAMM images were performed at 1.5 T prior to CRT and processed using a novel framework to extract regional ventricular volume-change, thickening and deformation fields (strain) [160]. A systolic dyssynchrony index (SDI) for all parameters within a 16-segment model of the ventricle was computed with high SDI denoting more dyssynchrony. 21 of 44 patients (48 %) recruited in the first phase (mean QRS duration 154 ± 24 ms) patients showed reverse remodelling (RR). Volume-change SDI was the strongest predictor of RR (PR 5.67; 95 % CI 1.95–16.5; *P* = 0.003). SDI derived from myocardial strain was least predictive. Volume-change SDI was applied as a predictor of RR to a second population of 50 patients (mean QRSd 146 ± 21 ms). When compared to QRSd, LBBB morphology and scar burden, volume-change SDI was the only statistically significant predictor of RR in this group. The authors conclude that volume-change SDI is a highly reproducible measurement that can be derived from routinely acquired SSFP cine images and predicts reverse remodelling following CRT.

## Coronary artery disease

CMR is invaluable to the study of patients with ischaemic heart disease and has made a major impact on this field and is now widely used to delineate myocardial infarction, [161, 162] and scarring, [163] although more recently non-contrast techniques to detect infarction have been described. The use of techniques to define myocardial edema, [164–166] myocardium at risk, [167] myocardial hemorrhage, and microvascular obstruction, [168] has also grown greatly. Work has moved on to evaluating the relation between myocardial damage and aspects of myocardial function such as dyssynchrony, remodelling, healing, and determining outcomes, [169] and the indication for novel treatments [170–172]. Major use of myocardial perfusion imaging by CMR has grown substantially, [173–175] including assessment of coronary flow reserve, [176, 177] and technical development [178–181]. The papers below show how CMR can be used in a number of ways to characterize acute and chronic coronary disease.

### Prediction of long-term segmental and global functional recovery of hibernating myocardium after revascularisation based on low dose dobutamine and late gadolinium enhancement cardiovascular magnetic resonance

This study evaluated the relation between baseline markers of viability and long-term functional outcome in 42 patients with ischaemic heart disease after revascularisation [182]. Both the likelihood and time course of functional improvement were related to LGE transmurality, contractile reserve on low-dose dobutamine (LDD), and degree of contractile dysfunction at baseline. Patients with ≥55 % viable segments significantly improved and experienced reverse LV remodelling. A combination of LDD-CMR and LGE-CMR is a simple and powerful tool for identifying which patients with impaired LV function will benefit from revascularisation.

### In vivo contrast free chronic myocardial infarction characterization using diffusion-weighted cardiovascular magnetic resonance

A recently developed in vivo diffusion weighted CMR (dwCMR) technique was compared with LGE in 11 pigs with chronic MI [183]. Apparent diffusion coefficient (ADC) maps were derived from three orthogonal diffusion directions and one non-diffusion weighted image. The study showed that ADC-derived infarct volume and location had excellent agreement with LGE. In conclusion, dwCMR has the potential to become a contrast-free alternative to LGE in characterizing chronic MI.

### Multiparametric cardiovascular magnetic resonance surveillance of acute cardiac allograft rejection and characterisation of transplantation-associated myocardial injury: a pilot study

This prospective longitudinal study evaluated the performance of multiparametric CMR for detecting acute cardiac allograft rejection (ACAR) and characterizing graft recovery following transplantation [184]. Significant improvements were seen in markers of graft structure and function (such as LV mass, native T1 and T2, ECV, circumferential strain, rest and stress myocardial blood flow) over time. However, none of the CMR parameters reached statistical significance to detect ACAR in the 5/22 patients with ACAR on endomyocardial biopsy.

### Prevalence and extent of infarct and microvascular obstruction following different reperfusion therapies in ST-elevation myocardial infarction

The authors sought to characterise microvascular obstruction (MVO) and infarct size (IS) in relation to the mode and timing of reperfusion in 94 patients with ST-Elevation Myocardial Infarction (STEMI) [185]. IS was smaller in the primary PCI and thrombolysis groups compared to the late PCI and rescue PCI groups. In the reperfused patient group, time to reperfusion, ischaemic area at risk and TIMI grade post-PCI were the strongest predictors of IS and MVO. MVO was not exclusive of reperfusion therapy and was primarily related to ischaemic time. These findings have important implications for clinical trials that use CMR to assess the efficacy of therapies aimed to reduce reperfusion injury in STEMI.

### Assessment of global myocardial perfusion reserve using cardiovascular magnetic resonance of coronary sinus flow at 3 Tesla

Despite increasing clinical use, there is limited data regarding regadenoson in stress perfusion CMR. Perfusion imaging was performed in 117 patients with suspected myocardial ischaemia and myocardial perfusion reserve (MPR) was estimated by coronary sinus flow measurements [186]. A subgroup of 41 patients was given aminophylline after the stress images were acquired. MPR was significantly underestimated 15 min after regadenoson injection indicating residual hyperemia, which was completely reversed after administration of aminophylline. These findings support the routine use of aminophylline when performing stress CMR with regadenoson.

### Comparative cost-effectiveness analyses of cardiovascular magnetic resonance and coronary angiography combined with fractional flow reserve for the diagnosis of coronary artery disease

CMR and fractional flow reserve (FFR) allow for a reliable assessment of ischaemia in combination with anatomical information provided by coronary angiography (CXA). The cost-effectiveness of these two strategies was compared [187]. CMR + CXA and CXA + FFR were equally cost-effective at a pretest likelihood of CAD of 62 % in Switzerland, 65 % in Germany, 83 % in the UK, and 82 % in the US. Below these thresholds, CMR + CXA showed lower costs per patient correctly diagnosed than CXA + FFR. These findings may help to optimize resource utilization in the diagnosis of CAD.

### Voxel-wise quantification of myocardial blood flow with cardiovascular magnetic resonance: effect of variations in methodology and validation with positron emission tomography

This study evaluated the effect of common methodological differences in CMR voxel-wise measurement of MBF, using position emission tomography (PET) as the reference standard [188]. Eighteen subjects (nine with coronary artery disease and nine healthy volunteers) underwent perfusion CMR. MBF was quantified using 1. Calculated contrast agent concentration curves (to correct for signal saturation) versus raw signal intensity curves; 2. Mid-ventricular versus basal-ventricular short-axis arterial input function (AIF) extraction; 3. Three different deconvolution approaches; Fermi function parameterization, truncated singular value decomposition (TSVD) and first-order Tikhonov regularization with b-splines. MBF was significantly higher when calculated using signal intensity compared to contrast agent concentration curves, and when the AIF was extracted from mid- compared to basal-ventricular images. Agreement between all deconvolution methods was high and MBF derived using each CMR deconvolution method showed a significant linear relationship with PET-derived MBF, however each method underestimated MBF compared to PET (by 0.19 to 0.35 mL/min/g).

### Susceptibility-weighted cardiovascular magnetic resonance in comparison to T2 and T2 star imaging for detection of intramyocardial hemorrhage following acute myocardial infarction at 3 Tesla

Detection of intramyocardial haemorrhage (IMH) by T2-weighted imaging or T2* may be limited by long breath holds and sensitivity to artefacts, particularly at 3 T. This paper evaluated susceptibility weighted imaging (SW MRI) for detection of IMH [189]. Forty-nine patients post-acute MI patients underwent CMR using T2-weighted, T2* and SW MRI. SW MRI had shorter breath hold times than either T2-weighted or T2*, with comparable diagnostic accuracy and inter-operator reliability to T2-weighted imaging and superior to T2*. SW MRI therefore provides an accurate and reproducible method for imaging IMH with considerably shorter breath hold times to conventional imaging.

## Pacemakers

With the approval of some makes of pacemakers and implantable defibrillators for use in the MR scanner, there has been considerable interest in reporting their use and safety, [190, 191] and clinical services are expanded significantly.

### Safe performance of magnetic resonance of the heart in patients with magnetic resonance conditional pacemaker systems: the safety issue of the ESTIMATE study

The purpose of this study was to analyse safety and potential alterations of electrical lead parameters in 36 patients implanted with the EnRhythm/Advisa MRI SureScan PM during and after CMR at 1.5 Tesla [192]. No adverse events occurred during CMR or thereafter. Statistically significant changes were noted on ventricular sensing, impedance and pacing capture threshold after the CMR scan albeit clinically irrelevant. Hence the results suggest CMR to be safe in patients with the MR conditional EnRhythm/Advisa system.

### Immediate and 12 months follow up of function and lead integrity after cranial MRI in 356 patients with conventional cardiac pacemakers

This study evaluated the safety of conventional cardiac pacemakers undergoing cranial MRI at 1.5 Tesla [193]. A total of 356 scans were completed without complications. No arrhythmias were induced, programmed parameters remained unchanged, and no pacemaker dysfunction was identified. Follow-up examinations performed up to 12 months after the scan did not show any significant change of pacing capture threshold, sensing threshold or lead impedance. This supports the evidence that patients with conventional pacemakers can safely undergo cranial MRI in a 1.5 T system with suitable preparation, supervision and precautions.

## Varia

There are always papers which do not fall into simple categories. This section pulls together such varia which has included interventional CMR techniques, [194–196] reviews, [197–199] cost-effectiveness, [200] clinical decision making, [201] society reports, [202–204] corrections, [205] registries, [206–208] and novel techniques [209, 210].

### Simplifying cardiovascular magnetic resonance pulse sequence terminology

The paper presents recommendations for unified terms to describe CMR pulse sequences based on the purpose of each sequence rather than the technical approach [211]. As well as standardizing terminology between vendors, this may improve written communication between CMR-requesting clinicians and CMR readers. For example, T2 weighted sequences would be referred to as “edema CMR” and T2* as “iron CMR”. For research articles, use of both simplified and more technical terms would allow the methods to be more accessible to non-expert readers.

### Use of oral gadobenate dimeglumine to visualise the oesophagus during magnetic resonance angiography in patients with atrial fibrillation prior to catheter ablation

The authors describe a method where an oral gel solution of 0.7 mL gadobenate dimeglumine contrast was swallowed by patients immediately before a free-breathing 3D MRA acquisition [212]. The oesophagus was visualized in 104/105 patients. All patients tolerated the study protocol and no complications were observed. Oesophagus visualization with an oral gadolinium solution is feasible for integration of its anatomy alongside the left atrium and pulmonary veins into an electro-anatomical map. The proposed strategy may potentially reduce the risk of atrio-oesophageal fistulae during AF radiofrequency ablation.

### Vortex flow during early and late left ventricular filling in normal subjects: quantitative characterization using retrospectively-gated 4D flow cardiovascular magnetic resonance and three-dimensional vortex core analysis

Imaging of LV diastolic vortex formation may allow valuable insights in evaluation of LV function and measures of LV filling efficiency. 4D Flow CMR was acquired in healthy volunteers during passive (E) and active (A) diastolic filling using retrospective cardiac gating and 3D vortex analysis [213]. The orientation of the vortex ring core and ring shape were quantified. The centre of the A-vortex was significantly closer to the mitral valve annulus and more elliptical in shape compared to the E-vortex. There was a strong correlation between the shape of the vortex and the mitral inflow shape through the annulus and leaflet tips. Normal values in a cohort of healthy volunteers are reported.

### Tracking of stem cells in vivo for cardiovascular applications

Stem cells for regeneration of the myocardium has received much attention over the past 10 years, with conflicting trial data regarding efficacy. This review focuses on the role of CMR in assessment of myocardial regeneration and for confirmation of successful cell delivery and engraftment [214]. Cellular labeling techniques and their application in CMR and ultrasound are discussed and trial data to date is reviewed.

### Adenosine stress native T1 mapping in severe aortic stenosis: evidence for a role of the intravascular compartment on myocardial T1 values

The group assessed the impact of the intravascular compartment on native T1 values in a cohort of patients with severe aortic stenosis studied at rest and adenosine-induced hyperaemia [215]. As expected, patients had increased myocardial T1 at rest compared to controls, however, during adenosine stress, T1 values were similar between AS and controls. Repeat T1 several months after aortic valve replacement demonstrated a resting and hyperemic T1 that had recovered towards normal. Native T1 values are therefore influenced by changes in the intravascular compartment as well as interstitial and intracellular fluid changes.

## Abbreviations

MA: Mitral annulus
MBF: Myocardial blood flow
MDCTA: Multi-detector computed tomography angiography
MI: Myocardial infarction
MRA: Magnetic resonance angiography
MVO: Microvascular obstruction
PCA: Principal component analysis
PDA: Patent ductus arteriosus
PET: Positron emission tomography
PH: Pulmonary hypertension
PWV: Pulse wave velocity
RAD: Renal artery stenosis
RV: Right ventricle
SCD: Sudden cardiac death
STEMI: ST elevation myocardial infarction
TAVI: Transcatheter aortic valve implantation
ToF: Tetralogy of Fallot
TSA: Turbo spin echo
TTE: Transthoracic echocardiography
